# SAX-HPLC and HSQC NMR Spectroscopy: Orthogonal Methods for Characterizing Heparin Batches Composition

**DOI:** 10.3389/fmed.2019.00078

**Published:** 2019-04-18

**Authors:** Franco Spelta, Lino Liverani, Alessandra Peluso, Maria Marinozzi, Elena Urso, Marco Guerrini, Annamaria Naggi

**Affiliations:** ^1^R&D Department, Opocrin S.p.A., Formigine, Italy; ^2^Istituto di Ricerche Chimiche e Biochimiche “G. Ronzoni”, Milan, Italy

**Keywords:** heparin, characterization, composition, building blocks, SAX-HPLC, quantitative NMR, HSQC

## Abstract

Heparin is a complex mixture of heterogeneous sulfated polysaccharidic chains. Its physico-chemical characterization is based on the contribution of several methods, but advantages of the use of complementary techniques have not been fully investigated yet. Strong-Anion-Exchange HPLC after enzymatic digestion and quantitative bidimensional ^1^H-^13^C NMR (HSQC) are the most used methods for the determination of heparin structure, providing the composition of its building blocks. The SAX-HPLC method is based on a complete enzymatic digestion of the sample with a mixture of heparinases I, II and III, followed by the separation of the resulting di- and oligo-saccharides by liquid chromatography. The NMR-HSQC analysis is performed on the intact sample and provides the percentage of mono- and di-saccharides by integration of diagnostic peaks. Since, for both methods, accuracy cannot be proved with the standard procedures, it is interesting to compare these techniques, highlighting their capabilities and drawbacks. In the present work, more than 30 batches of porcine mucosa heparin, from 8 manufacturers, have been analyzed with the two methods, and the corresponding results are discussed, based on similarities and differences of the outcomes. The critical comparison of both common and complementary information from the two methods can be used to identify which structural features are best evaluated by each method, and to verify from the concordance of the results the accuracy of the two methods, providing a powerful tool for the regular characterization of single, commercial preparations of Heparin.

## Introduction

Heparin is the most important anticoagulant drug and has been used in clinical practice since 1939. Although heparin was discovered nearly a 100 years ago, its structure/function relationships are still the subject of many studies (1). What makes investigations on the interaction of heparin with biologic systems very difficult is that, unlike most biological substances, heparin has an “intrinsically variant” structure. The structure of heparin chains is based on disaccharide building blocks, all made up of a uronic acid and a glucosamine. Variations in the composition of heparin include distinct uronic acid content, i.e., different glucuronic to iduronic ratios (GlcA/IdoA), different levels of N-acetylation/N-sulfation of the glucosamine and of 2-O- and 6-O-sulfation (in iduronate and glucosamine, respectively), as well as variable degrees of 3-O sulfation in glucosamine (2–4). The industrial processes of extraction and purification of heparin can cause further variability, affecting its structure in many points, and at different levels (“process signatures”), causing partial chemical modifications such as: depolymerization, N-desulfation (5), O-desulfation with epoxidation and/or epimerization on C2-C3 of sulfated iduronic acids (6), oxidation and/or disruption of the linkage region (LR) (7, 8) and other minor defects (9–13). Currently, the only source of heparin preparations in US, Europe and Japan is porcine mucosa, but bovine and ovine heparin preparations are available in other countries, and this might be another cause of variability, as each source shows a typical set of characteristic features (14–16). The most evident differences that can be observed among heparin from different sources are related to the sulfation pattern: e.g., porcine heparin is largely sulfated in position 6 of glucosamine, in comparison to bovine mucosa heparin, whereas the 2-O sulfation of iduronic acid is higher in bovine mucosa heparin than in porcine (4).

A large number of analytical methods have been used to address the high variability of heparin structure (17), but only two of them are practical enough and, at the same time, able to provide a sufficiently detailed and precise description of the oligomeric composition of commercial batches: building block analysis by Strong-Anion-Exchange HPLC after exhaustive enzymatic digestion (SAX-HPLC) (18) and quantitative NMR Heteronuclear-Single-Quantum-Coherence experiments (HSQC) (19).

The SAX-HPLC method involves the enzymatic cleavage of the heparin sample into its building blocks (mainly disaccharides): a mixture of Heparinases I, II, and III cleaves the linkage between the glucosamine and the uronic acid introducing a double bond in position C4-C5 (“Δ”) of the uronic acid. The mixture of di- and oligo-saccharides is resolved on a chromatographic system, where the new double bond makes it possible to detect all these saccharides by UV at 234 nm: a simple calculation provides a molar ratio of each building block. According to a general consensus, based on some papers (18, 20) the same molar absorption coefficient for all Δ4-5 unsaturated heparin di- and oligosaccharides has been used. As no clear evidence of this sameness has been provided up to now, an independent assessment of the response factor of the nine Δ4-5 unsaturated disaccharides, available on the market as reference materials, was an additional aim of this work.

The enzymatic cleavage causes the loss of information about the epimerization of the uronic acids (iduronic or glucuronic), which are no longer distinguishable. Moreover, some sequences along the heparin chain (e.g., disaccharides made up of a glucosamine with a sulfate in position 3, or the whole linkage region) are not affected by the enzymatic activity resulting in the formation of tetrasaccharides (21).

In the HSQC method the monosaccharides and disaccharides composition (molar ratio) is calculated by normalizing volumes of the signals of the 2-D NMR spectrum with reference to the sum of volumes of signals corresponding to the same monosaccharide type (glucosamines or uronic acids) and the same carbon-proton pair type (e.g., anomeric proton carbon pairs or C2 ring position pairs). Details of the method can be found in published papers, where calculation formulas, the influence of key experimental parameters and validation results are described (19, 22). The main advantage of the HSQC method is that no sample manipulation is required before the analysis and that iduronic and glucuronic acid residues can be distinguished and quantified. In contrast, information about which type of glucosamine residue is sulfated or not in position 6 is not available and only the overall amount of 6-O-sulfation can be determined. A comparison of advantages and drawbacks of the two methods is shown in Table 1 (23).

**Table 1:** Major advantages and drawbacks of the two methods.

	**Advantages**	**Drawbacks**
**SAX-HPLC**	Di- and tetra-saccharide composition: single building blocks identified and quantified	Digestion with a mix of Heparinases: the thorough yield of the depolymerization reaction should be confirmed (e.g., by Size Exclusion Chromatography)
	High Sensitivity: LOD 0.1%, LOQ 0.3% (for disaccharides that respond to the mixture of Heparinases)	Sequences with specific process signatures not cleaved by the enzymes
	Identification of specific disaccharides containing 6-O-sulfated glucosamine	Iduronic/glucuronic structure not distinguished. Information only about uronic acid-glucosamine sequences
	Easily achievable in every analytical lab; standard equipment	Saturated residues at the non-reducing end of heparin chains not detectable
		Quantification based on consensus assumption that all Δ4-5 unsaturated di- and oligo-saccharides have the same molar absorption coefficient
**HSQC-NMR**	No sample treatment necessary: information about the overall structure	Low sensitivity: specific for each residue. LOD 0.5%, LOQ 2% on average
	Mono- and di-saccharide composition	Possible problems with signals resolution
	Iduronic and glucuronic acids can be distinguished	Quantification possible only by comparison of atoms with similar magnetic properties
	Information about both uronic acid-glucosamine and glucosamine-uronic acid sequences	Only the overall 6-Osulfation of glucosamine residues can be determined^*^

* The possibility of differentiating 6-O-sulfated and non-sulfated glucosamine by 1D proton NMR has been recently described (23). However, the resolution of the HSQC spectrum does not make it possible to resolve these peaks, if not at a very high magnetic field.

SAX-HPLC and HSQC methods are simple and can be standardized, providing a high amount of complementary, useful and quantitative information for the quality control of commercial batches of heparin. Both methods provide molar percentage distributions of heparin building blocks, but the two methods resolve and identify different fragments (Tables 2, 3).

**Table 2:** Heparin building blocks identified by the SAX-HPLC method of the present study: relative retention time with respect to the main disaccharide ΔIs, peak 14.

**peak ID**	**Building block code**	**Building block structure**	**Relative retention time**	**Residue identified by:**
1	L.R.	ΔGlcA-Gal-Gal-Xyl-Ser	0.190	Isolation through semi-prep. SAX-HPLC and LC/MS^£^
2	ΔIVa	ΔUA-GlcNAc	0.225	Comparison with standard disacch.
3	L.R., ox1	ΔGlcA-Gal-Gal-Xyl-CH_2_COOH^#^	0.405	isolation through semi-prep. SAX-HPLC and LC/MS^£^
4	ΔIVs_gal_	ΔGalA-GlcNS^$^	0.465	Isolation through semi-prep. SAX-HPLC and LC/MS^£^
5	ΔIVs	ΔUA-GlcNS	0.475	Comparison with standard disacch.
6	ΔIIa	ΔUA-GlcNAc,6S	0.520	Comparison with standard disacch.
7	ΔIIIa	ΔUA,2S-GlcNAc	0.585	Comparison with standard disacch.
8	ΔIh	ΔUA,2S-GlcN,6S	0.640	Comparison with standard disacch.
9	ΔIIs_gal_	ΔGalA-GlcNS,6S^$^	0.685	Isolation through semi-prep. SAX-HPLC and LC/MS^£^
10	ΔIIs	ΔUA-GlcNS,6S	0.700	Comparison with standard disacch.
11	ΔIIIs	ΔUA,2S-GlcNS	0.765	Comparison with standard disacch.
12	ΔIa	ΔUA,2S-GlcNAc,6S	0.880	Comparison with standard disacch.
13	ΔIIa-IVs_glu(3S)_	ΔUA-GlcNAc,6S-GlcA-GlcNS,3S	0.975	Comparison with published data (18)
14	ΔIs	ΔUA,2S-GlcNS,6S	1.000	Comparison with standard disacch.
15	ΔIIa-IIs_glu(3S)_	ΔUA-GlcNAc,6S-GlcA-GlcNS,3S,6S	1.085	isolation through semi-prep. SAX-HPLC and LC/MS^£^
16	ΔIIs-IIs_glu(3S)_	ΔUA-GlcNS,6S-GlcA-GlcNS,3S,6S	1.175	Comparison with published data (18)
17	ΔIs_glu(3S)_	ΔUA,2S-GlcNS,3S,6S	1.265	Comparison with published data (18)
18	ΔIa-IIs_glu(3S)_	ΔUA,2S-GlcNAc,6S-GlcA-GlcNS,3S,6S	1.300	Comparison with published data (18)
19	ΔIs-IIs_glu(3S)_	ΔUA,2S-GlcNS,6S-GlcA-GlcNS,3S,6S	1.360	Comparison with published data (18)

£ data not shown.

# “process signature”. Linkage region with an oxidized serine, due to heparin purification steps.

$ “process signature”. 2-O-desulfation of iduronic acid and its epimerization caused by strong alkaline processes and thermal stress.

*Underlinings remark disaccharides with a glucuronic acid and a galactosamine with an additional sulfate in position 3*.

**Table 3:** Heparin building blocks identified by the HSQC method.

**Glucosamine residues**	**Uronic acid residues**
GlcNH_2_,6x	GlcA-GlcNAc,6x
GlcNS,3S,6x	GlcA-GlcNS,6x
GlcNAc,6x-GlcA	GlcA-GlcNS,3S,6x
GlcNAc,6x-IdoA	GlcA,2S
GlcNS,6x-GlcA	IdoA-GlcNy
GlcNS,6x-IdoA	IdoA-GlcNy,6S
GlcNS,6x-IdoA,2S	IdoA,2S-GlcNH_2_,6x
GlcNS,6x-GalA	IdoA,2S-GlcNy,3x,6x
GlcNS,6x-Epox	GalA
GlcNAc,6x, αRed	Epox
GlcNS,6x, αRed	GlcA-Linkage Region
GlcNx,6S	

The precision of both methods is assured [Supplementary Table 1 for SAX-HPLC and (19) for HSQC] but, because no official reference material (a heparin sample with known composition) can be devised, accuracy of each separate method cannot be evaluated. Only the similarity of the results obtained on the same heparin sample by totally different methods can support their accuracy. Since the two methods do not provide information on the same residues, a direct comparison of all results is not possible. This work combines quantitative data of different residues in order to compare broader and more general attributes, as in the case of the level of sulfation on the different positions.

The present work is therefore aimed at providing a solid ground to the two methods, by comparing the results, looking for similarities and possible discrepancies for identifying the more reliable and accurate result in case of disagreement.

For this investigation, data from 33 Heparin Sodium batches, USP and/or Ph. Eur. compliant, from 8 manufacturers, covering a time span from 2011 to 2016, were collected and compared.

## Materials and Methods

### Samples

Thirty-three Heparin Sodium batches were selected from 87 samples of the updated “bona fide” library of the Ronzoni Institute (24), aiming to cover the highest number of heparin manufactures and the largest structural variability. All batches were USP and/or Ph. Eur. compliant. A list of all batches, with the relevant details, can be found in Supplementary Table 2.

Four additional, modified, heparin samples were prepared in Opocrin for investigating specific process signatures that are usually present at low levels in pharma grade heparin batches. Two batches with a high level of N-desulfation on glucosamine, and two batches with a high level of epoxide on the uronic acid were produced. Details about the production of these 4 batches are reported in section Production of heparin batches with higher content of specific features.

### Enzymatic Digestion

Heparin digestion was carried out by mixing 10 μL of a 10 mg/mL solution of sample in water with 20 μL of a calcium buffer (bovine serum albumin 0.1 mg/mL, calcium acetate 2 mM, sodium acetate 100 mM; pH 7.0) and 10 μL of a Heparinase I, II and III mixture, 0.25 IU each one, in phosphate buffer (bovine serum albumin 0.2 mg/mL, sodium phosphate 10 mM; pH 7.0). The mixtures were incubated for 48 h at room temperature for a complete digestion. Completeness of digestion was verified by a double check:

- The results of a Suitability sample, present in all analytical sets, were compared with past, historical results on a control chart.
- A selection of 15 digested samples were checked by Size Exclusion Chromatography: the absence of hexasaccarides or longer oligosaccharides, both by UV (234 nm) and refractive index detection, was considered sufficient to demonstrate the efficiency of the enzymatic digestion (details in Supplementary Text 1).

All reagents were of analytical grade and the three Heparinases were from CPC Biotech (Monza, Italy) or Grampian Enzymes (Aberdeen, UK). Water was from a Milli-Q purification system (Millipore).

### Strong-Anion-Exchange HPLC

**Figure 1:**
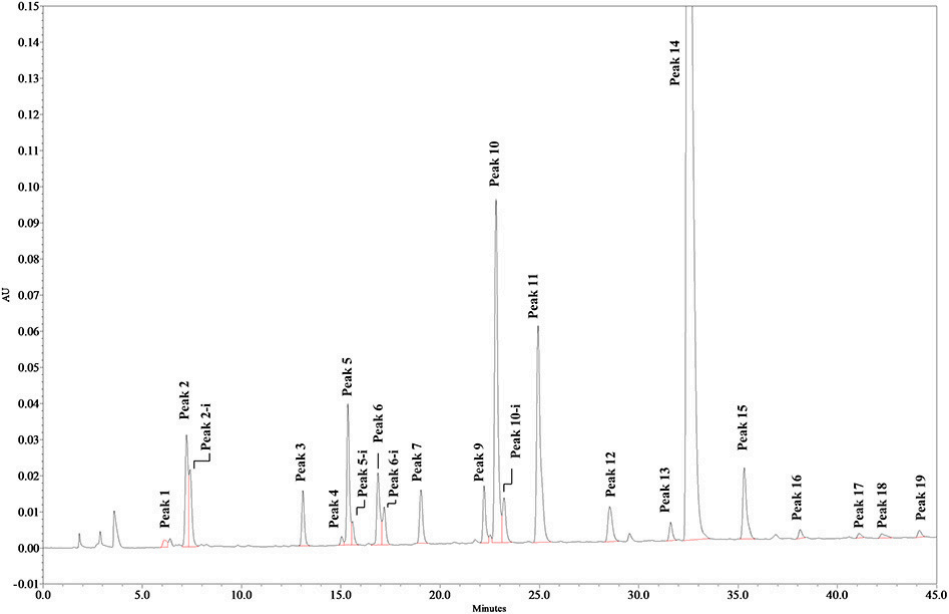
SAX-HPLC chromatogram of Heparin batch Man_D-2. Some disaccharides show a double peak, identified by the extension “-i,” depending on the elution of the two anomeric forms of the reducing end terminal. Early peaks, eluting in the range between 0 and 5 min, are system peaks. Other very small peaks could not be assigned, but no sample has shown an un-identified peak area larger than 0.25%, or a total area of un-identified peaks larger than 1.2%.

Solutions of digested heparin (10 μL) were injected onto a Spherisorb SAX column, 4.0 × 250 mm, 5 μm (Waters). The mixture of saccharides was resolved according to the chain length, the number of sulfates and their position by a linear gradient elution with mobile phase A (sodium phosphate 2 mM, pH 3.0) and B (sodium phosphate 2 mM, sodium perchlorate 1.0 M; pH 3.0): mobile phase B, t 0–0.5 min., 3%, t 20 min., 35%; t 50 min., 100%. Flow rate was 0.8 mL/min., with a column temperature of 40 °C and UV detection at 234 nm. Nineteen structures (12 di-, 5 tetra-, and 2 oligo-saccharides), all carrying unsaturated uronic acid at the non-reducing end (20) can be identified with this method: the 9 main, typical heparin disaccharides were identified by comparison of their retention time with that of pure disaccharides (Iduron; Alderley Edge, UK); 5 structures were isolated by semi-preparative SAX-HPLC and identified by LC/MS (data not shown), and 5 were identified by comparison with published data (18), in agreement with our findings. The list of the 19 oligosaccharide structures is shown in Table 2, in order of elution. A SAX-HPLC chromatogram, as an example, is shown in Figure 1.

All identified peaks were integrated, a cumulative area calculated, and a molar percentage distribution was obtained applying the same molar absorption coefficient (see section SAX-HPLC: building blocks and process signatures identified). The method was validated, and its main characteristics are summarized in Supplementary Table 1.

### Quantitative NMR Determination of the Typical Heparin Disaccharides and of Their SAX-HPLC Response Factor

The content of each vial of the 9 disaccharides (about 1 mg each, from Iduron, UK) was dissolved with 1.0 mL of D_2_O with TSP 0.002%. The molar concentration of these solutions was assessed by proton NMR.

The ^1^H-NMR spectra were recorded at 298 K on a 500 MHz Bruker AVANCE HD spectrometer equipped with a TCI cryoprobe, using the following parameters: number of scans 16, dummy scans 8, relaxation delay 25 s, spectral width 16 ppm, transmitter offset 4.7 ppm. After exponential multiplication (line broadening of 0.3 Hz), the spectra were Fourier-transformed, phased and baseline corrected. First, the absolute concentration of TSP (expressed in mol/g) was determined using a standard of potassium phthalate monobasic (KHP) certified for NMR (Sigma-Aldrich, product number 14659) as follows: about 20 mg of KHP was dissolved in 2.0 mL of TSP, 0.6 mL of the solution was transferred in a NMR tube and analyzed.

The following signals were integrated:

- The TSP signal at 0.00 ppm excluding the ^1^H-^13^C satellite peaks
- The aromatic signals of KHP at 7.57 (H3) and 7.72 ppm (H2), excluding the ^1^H-^13^C satellite peaks at 7.87 and 7.40 ppm; the other satellite peaks are superimposed to the signals of KHP.

The concentration of TSP was determined as follows:

$$\begin{array}{l} C_{TSP}=\frac{g_{KP}}{MW_{KHP}}\cdot \frac{I_{TSP}}{{0.995\;I}_{KHP}}\cdot \frac{4}{9}\cdot \frac{1}{g_{solvent}} \end{array}$$

*g*_*KP*_ and *MW*_*KHP*_ are the amount and the molecular weight of the potassium phthalate monobasic, respectively, *I*_*TSP*_ is the integration of the TSP signal, *I*_*KHP*_ is the sum of the integrations of the potassium phthalate monobasic signals, *g*_*solvent*_ is the amount of the solvent, 0.995 is the correction coefficient due to the overlap of 50 % of the ^1^H-^13^C satellite peaks of potassium phthalate monobasic signals, 4 and 9 are the number of hydrogen atoms of KHP and TSP, respectively.

To quantify the amount of disaccharides, two aliquots for each sample solution (250 μL) were analyzed.

The integration of the following signals was measured:

- The TSP signal at 0.00 ppm excluding the ^1^H-^13^C satellite peaks
- The ΔU4 signal of the disaccharide

The amount of disaccharides was calculated as follows:

$$\begin{array}{l} mg_{dis}=C_{TSP}\cdot \frac{I_{dis}}{I_{TSP}/9}\cdot mg_{solution}\cdot MW_{dis} \end{array}$$

*C*_*TSP*_ is the concentration of the TSP, *I*_*dis*_ and *I*_*TSP*_ are the integral of H4 of the disaccharide and the TSP signal, respectively, *mg*_*solution*_ is the amount of the solution, *MW*_*dis*_ is the molecular weight of the disaccharide, 9 the number of hydrogen atoms in the TSP (Supplementary Figure 1).

Different aliquots (2, 6, 10, and 14 μL) of the same solutions were injected on the same SAX-HPLC system used for the present study: the response factors of each disaccharide were calculated as the mean of the 4 ratios “peak area/injected amount.” A relative response factor, with reference to the disaccharide ΔIs, was calculated to make the comparison easier. Results are shown in Supplementary Table 3.

### HSQC

The ^1^H-^13^C-HSQC spectra were measured on a Bruker AVANCE III 600 MHz spectrometer equipped with a 5 mm TCI cryoprobe, using the Bruker hsqcetgpsisp2.2 pulse sequence according to the published method (19). Briefly, the spectra were recorded at 298K using the following acquisition parameters: number of scans 12, dummy scans 16, relaxation delay 2.5 s, spectral width 8 ppm (F2) and 80 ppm (F1), transmitter offset 4.7 ppm (F2) and 80 ppm (F1), ^1^J_C−H_ = 150 Hz. 1024 points were recorded for each of 240 increments (NUS of 75 % of 320 increments). The FIDs were processed as follows: spectrum size 4096 (F2) and 1024 (F1) (zero-filling in F2 and linear prediction in F1), squared cosine window multiplication in both dimensions and Fourier-transform. The diagnostic heparin building block signals were integrated using Topspin software version 3.5 (Bruker BioSpin, Rheinstetten, Germany) and the heparin composition was computed from the integral values as previously described (19). The list of the 23 heparin features identified and quantified with this method is shown in Table 3.

### Production of Heparin Batches With Higher Content of Specific Features

#### Non N-Sulfated, Non N-Acetylated Glucosamine

Two batches were produced from the same parent heparin batch: two samples of ~3 g of heparin were dissolved separately with ~26.6 mL of water. The solutions were heated at ~50°C, and 3.4 mL of HCl 4M were added to each one. The solutions were maintained at ~50°C for 1 and 3 h, respectively. Each solution was neutralized with NaOH 4M, cooled at room temperature, and the product precipitated with ~78 mL of methanol. The precipitate was recovered by centrifugation and dissolved with ~60 mL of water; the volume was reduced to ~30 mL on a rotary evaporator and the samples were freeze-dried, giving origin to batches N-deS-1 and N-deS-2, respectively.

#### Uronic Acid, 2,3-epoxide

Two batches were produced from the same parent heparin of the previous samples: two samples of ~3 g of heparin were dissolved separately with ~27 mL of water. The solutions were heated at ~50°C, and 3.0 mL of NaOH 4M were added to each one. The solutions were maintained at ~50°C for 1 and 2.5 h, respectively. Each solution was neutralized with HCl 4M, cooled at room temperature, and the product precipitated with ~78 mL of methanol. The precipitate was recovered by centrifugation and dissolved with ~60 mL of water; the volume was reduced to ~30 mL on a rotary evaporator and the samples were freeze-dried, giving origin to batches epox_1 and epox_2, respectively.

## Results

### SAX-HPLC: Building Blocks and Process Signatures Identified

A SAX-HPLC chromatogram, as an example, is shown in Figure 1, while a table with individual results can be found in Supplementary Table 4: as previously reported, 19 oligosaccharide structures have been identified with the SAX-HPLC method of the present study (Table 2).

Among these 19 structures, one is the “linkage region,” the oligosaccharide that links the heparin chain to the protein core of the parent proteoglycan (peak 1), 8 are the disaccharides present in higher proportions, available as isolated reference materials (peaks 2, 5, 6, 7, 10, 11, 12, and 14) and one is the disaccharide ΔUA,2S-GlcNS,3S,6S (peak 17), which is the only disaccharide containing a sulfate in position 3 of the glucosamine unit that can be obtained by enzymatic digestion. This tetra-sulfated disaccharide is originated by the enzymatic cleavage of the -IdoA2S-GlcNS,3S,6S- sequence, which can be present in the heparin chain, or as an isolated disaccharide, or in the pentasaccharide sequence containing an additional 3-O-sulfated glucosamine (GlcNS,6S-GlcA-GlcNS,3S,6S-IdoA2S-GlcNS,3S,6S) (25), or in antithrombin binding sequences recently synthetized by chemoenzymatic methods, that might also be present in natural sequences (GlcNS,6S-GlcA-GlcNS,6S-IdoA2S- GlcNS,3S,6S-IdoA2S-GlcNS,6S-) (26).

Five residues are tetrasaccharides which were not cleaved to disaccharides by the enzymes, due to the presence of glucuronic acid followed by a 3-O-sulfated glucosamine: these tetrasaccharides make up part of the pentasaccharide variants responsible for the binding of heparin with antithrombin and, as a consequence, for its anticoagulant properties (peaks 13, 15, 16, 18, 19) (27).

The remaining 4 structures are “process signatures” derived from heparin extraction/purification processes, which make use of strong oxidizing reagents, basic and acid pHs, and high temperatures. One is related to an oxidized derivative of the linkage region (peak 3) (6), two are related to the formation of galacturonic acid (peaks 4 and 9) and the last one to the disaccharide resulting from a N-desulfation of the glucosamine (peak 8). The galacturonic acid is originated by alkaline and heat treatments occurring during the heparin purification process: alkalis cause the elimination of sulfate in position 2 of the uronic acid residue, producing a 2–3 epoxide. This structure can remain on the heparin chain, but the concurrent or following heat treatments can cause it to open, with inversion of the configuration of carbon 2 and 3, turning the L-iduronic into L-galacturonic acid (6). The N-desulfated glucosamine residue is mainly due to pH and thermal stress of heparin during its manufacturing process, even if a small amount of this disaccharide can be the natural marker of an incomplete biosynthesis (5).

The SAX-HPLC results (Supplementary Table 4) and the following considerations are based on the general consensus assumption that response factors at 232-234 nm for all Δ4-5 unsaturated oligosaccharides are the same (18, 20). A specific, concurrent study has been carried out to assess the actual response factors of the 9 heparin disaccharides available as reference materials (section Quantitative NMR determination of the typical heparin disaccharides and of their SAX HPLC response factor). This study demonstrates that the molar absorption coefficients are very similar and only a few minor disaccharides, present in heparin in small amounts, show small differences (Supplementary Table 3). The differences noted were considered small enough to justify the use of the same response factors for all the peaks of the SAX-HPLC chromatograms.

### HSQC

**Figure 2:**
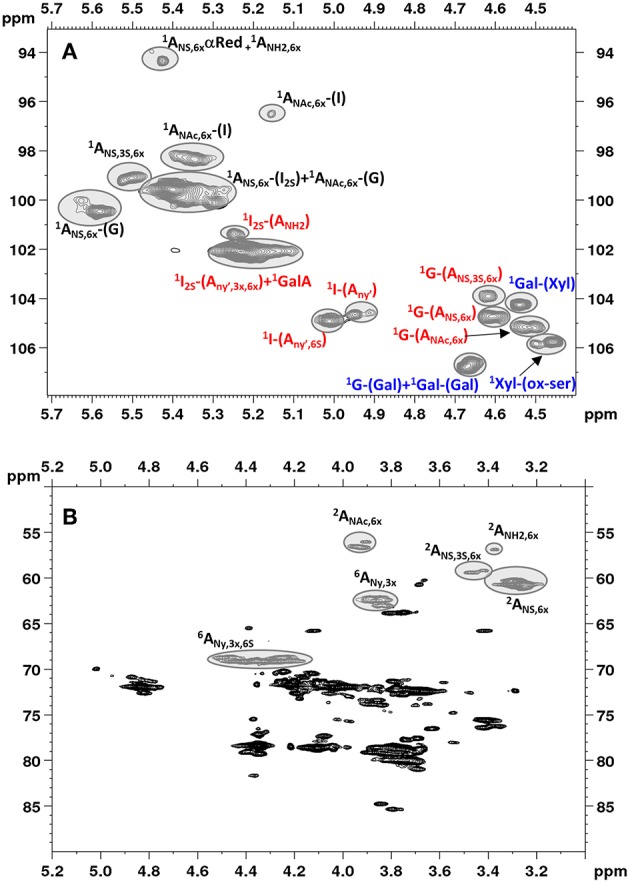
Low field region showing anomeric signals **(A)** and high field region **(B)** of HSQC spectrum of Heparin batch Man_D-2 with relevant signal assignments. Ovals identify the integrated signals. Glucosamine, iduronic acid and glucuronic acid residues are indicated as A, I and G, respectively. X = H or ${\text{SO}}_{3}^{-}$; Y = H or Ac or ${\text{SO}}_{3}^{-}$; Y' = Ac or ${\text{SO}}_{3}^{-}$.

Figure 2 shows an example of HSQC spectrum of a heparin sample: the signals used for quantitative analysis of individual uronic acid and glucosamine residues are highlighted. The list of the monomeric and disaccharidic structures—that could be identified with the HSQC method—is shown in Table 3, while individual results are shown in Supplementary Table 5. The building block composition and the percentage of 6-O-sulfation can be obtained directly by both HSQC and SAX-HPLC, but information about the differently substituted uronic acid residues (both glucuronic and iduronic) and the aminosugar-uronic acid sequences are obtained only with HSQC. It is worth noting that the results obtained by HSQC correspond to the molar percentage of each residue compared to the total amount of the corresponding monosaccharide type (i.e., glucosamine or uronic acid).

### Clustering of Data From SAX-HPLC and HSQC-NMR

The specific heparin features identified by the two methods—showed in Tables 2, 3—are not the same, as previously discussed. However, data and outcomes obtained by each method can be clustered according to homogeneous rules to provide comparable information on more general characteristics of the sample. By this process, data from the two methods can be evaluated by looking for similarities or discrepancies between the outcomes. The comparison of these data has been used to provide information on the accuracy of the two methods and has allowed more rational investigations on the possible discrepancies to be carried out.

Eleven heparin attributes have been found suitable for this clustering exercise: 7 related to the regular structure of heparin and 3 to possible process signatures, all reported as percentages. An additional attribute, the ratio sulfate to carboxylate ions (or “degree of sulfation”), is reported as a pure number. The list of these attributes, with a concise description of their meanings, is shown in Table 4. The way the single data are combined to originate information about each attribute can be found in Supplementary Table 6.

**Table 4:** List of the 11 heparin attributes that can be quantified by both the SAX-HPLC and HSQC methods, combining information from the single, raw, data.

GlcNS	Regular structure	Content of N-sulfated glucosamine
GlcNAc	Regular structure	Content of N-acetylated glucosamine
GlcNx,6S	Regular structure	Content of glucosamine with a sulfate in position 6
GlcNS,3S,6x	Regular structure	Content of a N-sulfated glucosamine with an additional sulfate in position 3
GlcA- GlcNS,3S,6x	Regular structure	Content of a disaccharide made up of a glucuronic acid and a glucosamine with an additional sulfate in position 3 (GlcA-GlcNS,3S,6x, typical of the “pentasaccharide” feature)
GlcNH_2_	Process signature	Content of glucosamine, non N-sulfated, non N-acetylated; N-desulfation due to pH and thermal stresses. Possible natural marker of an incomplete biosynthesis
IdoA,2S	Regular structure	Content of iduronic acid with a sulfate in position 2
GalA	Process signature	Content of L-galacturonic acid, due to the 2-O-desulfation of iduronic acid and the following opening of the epoxide, with inversion of configuration
Epox	Process signature	Content of uronic acid with residual epoxide in C2-C3, not opened by further steps of heparin processes
Linkage region (LR)	Mixed information	The SAX-HPLC method identifies only two major species: “native” LR and one oxidized species; the HSQC method detects all glucuronic acids linked to a galactose, i.e., all “native” and oxidized species

### Comparison of Results

**Figure 3:**
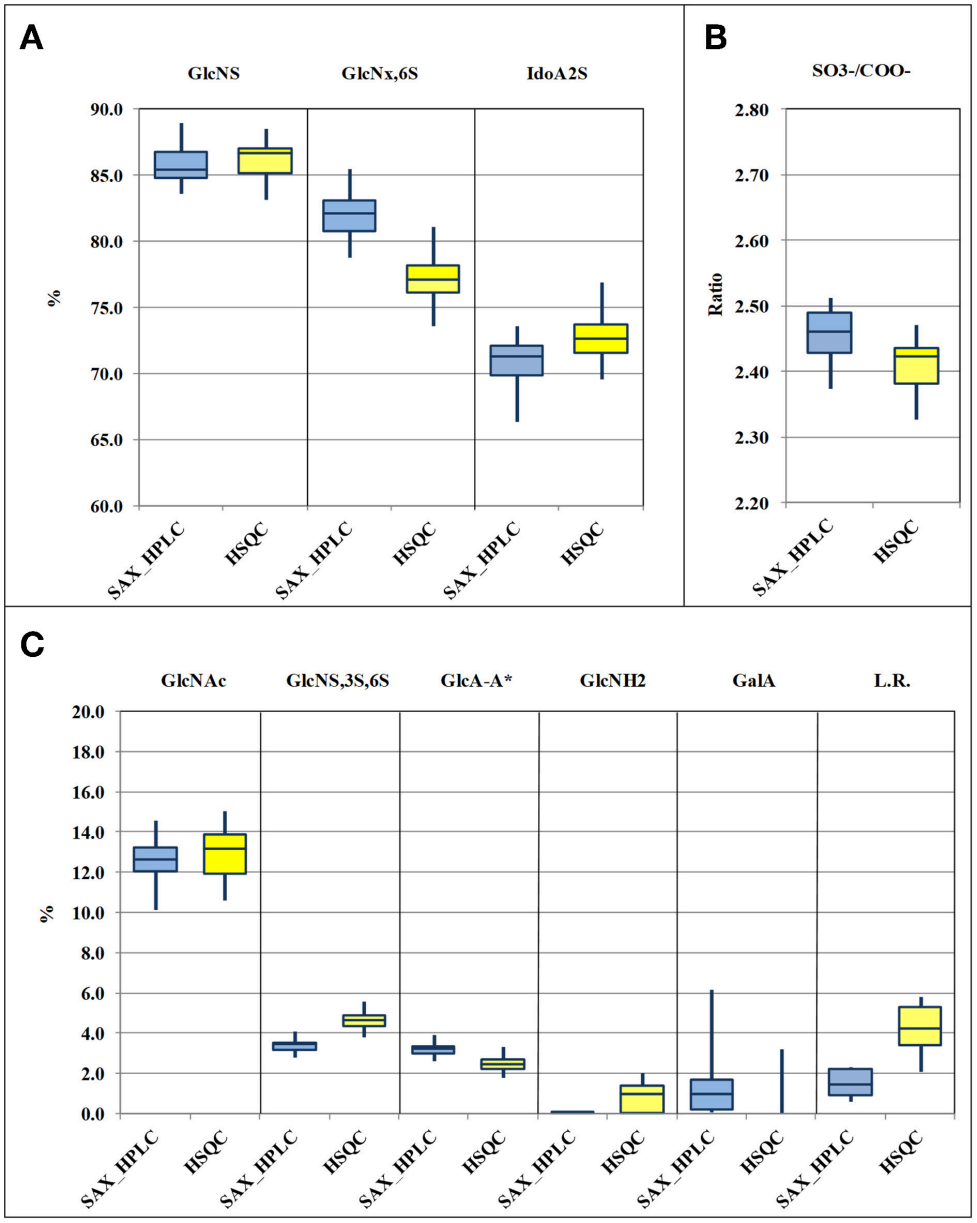
Box-plots (median, first and third percentiles, range) of the main heparin attributes of the 33 heparin batches : N-sulfated glucosamine (GlcNS), 6-O-sulfated glucosamine (GlcNx,6S) and 2-O sulfated iduronic acid (IdoA2S) **(A)** molar ratio of sulfate to carboxylate ions (${\text{SO}}_{3}^{-}$/COO^−^) **(B)** N-acetylated glucosamine (GlcNAc), GlcA-A^*^ or 3-O-sulfated-glucosamine (GlcNS,3S,6x), glucuronic acid linked to 3-O-sulfated-glucosamine (GlcA-GlcNS,3S,6x), N-desulfated glucosamine (GlcNH_2_), galacturonic acid (GalA) and linkage region (LR) **(C)**.

Results obtained by the two methods, for the 33 heparin batches and for the 11 attributes, following the required calculations, are shown in Supplementary Table 7 and are summarized and compared in the box-plots of Figure 3. Data relevant to the content of epoxide on uronic acid are not shown in this table, as explained below. The comparison of the box-plots of Figure 3A (SAX-HPLC vs. HSQC) shows that the two methods detect similar contents of the main heparin attributes: N-sulfated glucosamine, N-acetylated glucosamine, 6-O-sulfated glucosamine and iduronic acid with a sulfate in position 2. The agreement of the two methods for these attributes is confirmed by similar results of the molar ratio of sulfate to carboxylate ions (Figure 3B), which summarizes the previous attributes.

**Figure 4:**
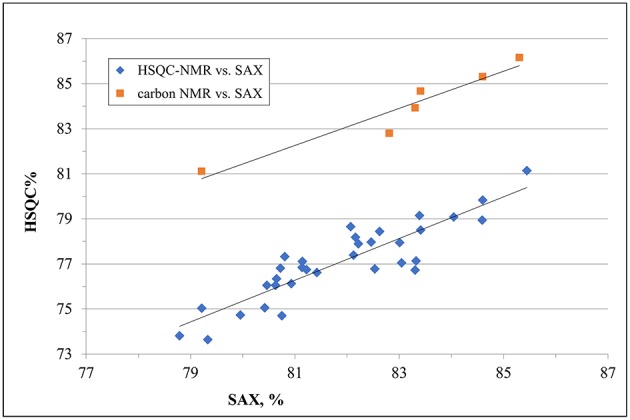
Comparison of results for the content of glucosamine with a sulfate in position 6 (GlcNx,6S): results from SAX-HPLC vs. HSQC (blue dots) and ^13^C-NMR (red squares). The distribution of blue dots shows a clear correlation between the content of 6-sulfated glucosamine from the two methods, where results from the SAX method are always higher than those from the HSQC. Quantitative results from the carbon spectra of a sub-set of 6 heparin samples (red squares) show a much better agreement with SAX results, confirming a greater accuracy of the enzymatic method than the HSQC.

Despite a general agreement of results from the two methods, a more accurate analysis of the data shows that some small differences can be detected. Glucosamine residue with a sulfate in position 6 (GlcNx,6S in Table 4 and Supplementary Table 6) shows a first difference: figures from the SAX-HPLC method are always higher, with a clear correlation between data from the two methods (Figure 4, blue dots). The reason for this systematic difference was investigated, and a longer spin-spin relaxation time of protons (T2) of non-sulfated C6 compared to sulfated C6 was considered responsible for unbalanced outcomes for C6 of GlcNx,6S and GlcNx,6OH signal volumes. The lower T2 of GlcNx,6S induces a higher loss of transverse magnetization during the pulse sequence compared to that of GlcNx,6OH, with a consequent underestimation of the residue GlcNx,6S, according to Mauri et al. (19). This problem was investigated on a subset of 6 heparin samples, expected to cover the whole range of values from the HSQC method, with additional ^13^C-NMR spectra, where the proton T2 effect is negligible. The results obtained from carbon spectra are, in this case, very close to those obtained by the SAX-HPLC method (Figure 4, red squares and Supplementary Table 8). According to these data, more accurate information on the content of glucosamine sulfated in position 6 can be obtained from the SAX-HPLC method.

Also figures of the content of 2-O-sulfated uronic acid are similar, but values from the SAX-HPLC method are always a bit lower, with a large scattering of the differences around a value of −2.0%: a mix of random and systematic errors was suspected. One point is that SAX-HPLC figures are obtained by the sum of 7 peaks, while HSQC figures are obtained by the sum of only two signals (three if the signal of 2-sulfated glucuronic acid is higher than its LOD): this imbalance might increase the scattering of the differences. On the other hand, a systematic error can be due to a problem with the HSQC method: the anomeric signal of galacturonic acid (GalA in Table 4) is embedded in that of 2-sulfate iduronic acids. The HSQC method tries to remove the GalA contribution by using the H5/C5 signal of GalA instead of the anomeric signal. Unfortunately, as made clear in a point below, this signal is always underestimated, so that the final adjustment does not prove to be fully correct. Batches with a higher content of GalA are more affected by this problem and show slightly overestimated values of IdoA2S by the HSQC method (Supplementary Table 7).

The molar ratio of sulfate to carboxylate ions resumes all the main attributes of heparin, and the comparison of figures from the two methods shows that they are very similar, with values from HSQC just slightly lower than those from the SAX-HPLC method (Figure 3B). This small difference is clearly in agreement with the larger effect of the underestimation of the content of glucosamine 6-sulfated by the HSQC method (Figure 3A). According to these findings, slightly more accurate information on sulfate to carboxylate ions ratio can be obtained from the SAX-HPLC method.

The content of 3-O-sulfated glucosamine and of the disaccharide glucuronic acid linked to 3-O-sulfated glucosamine (GlcNS,3S,6x and GlcA-GlcNS,3S,6x, respectively; Table 4) are important attributes of heparin, as these structures are somehow related to the content of the antithrombin binding sequence responsible for most of its anticoagulant properties (28). The HSQC method can measure both the disaccharide GlcA-GlcNS,3S,6x (by means of the signal of glucuronic acid linked to the GlcNS,3S,6x residue) and the total amount of the monosaccharide GlcNS,3S,6x, directly from its corresponding C1 or C2 signals, respectively. On the contrary, the SAX-HPLC method cannot provide the content of the monosaccharide GlcNS,3S,6x, but only of the disaccharide ΔIdoA2S-GlcNS,3S,6S (peak 17 in Table 2) and of the tetrasaccharide variants containing the disaccharide GlcA-GlcNS,3S,6x (peaks 13, 15, 16, 18, and 19 in Table 2). The combined information is used to provide data on the content of the disaccharide GlcA-GlcNS,3S,6S and to estimate the content of the monosaccharide GlcNS,3S,6S. The content of the disaccharide GlcA-GlcNS,3S,6x obtained by SAX-HPLC (Figure 3C) is significantly higher compared to HSQC. A possible reason for the lower levels detected by the HSQC method is that some sequence effects can generate weak and in part undetectable signals in the HSQC spectrum. On the other hand, the higher content of GlcNS,3S,6x found by the HSQC method can be explained by the presence in heparin preparations of 3-O-sulfated glucosamine at the non-reducing end of the chains, recently described by some authors (29, 30). This monosaccharide can be cleaved from the non-reducing end by the enzymes in the SAX-HPLC method, producing a saturated GlcNS,3S,6x residue, unnoticeable by UV detection. Therefore, more accurate information on the content of the monosaccharide GlcNS,3S,6x can be obtained from HSQC, while more accurate information on the content of the different sequences containing the disaccharide GlcA-GlcNS,3S,6x can be obtained by SAX-HPLC.

Data comparison shows a difference between the two methods also for GlcNH_2_ residue, a “process signature” affecting the glucosamine residue (ΔIh, peak 8 in Table 2 and GlcNH_2_ in Table 4) which is usually generated by uncontrolled pH and thermal stresses during heparin manufacturing. This feature is a major and important stability indicating attribute for heparin formulations as well (31). Results in Supplementary Table 7 show that the SAX-HPLC method was unable to detect the disaccharide ΔIh (ΔUA,2S-GlcNH,6S) in 29 out of the 33 heparin batches. Only 4 batches showed a content of ΔIh close to the limit of detection (0.1%). On the contrary, the HSQC method detected the GlcNH_2_ in almost all heparin batches, in a range between 0.7 and 2.1 %, when the limit of detection is close to 1%. The reasons for this difference were investigated with specific experiments: a heparin sample was deliberately over-stressed with a treatment with HCl 0.45 M, for 1 and 3 h at 50°C (subsection Non N-sulfated, non N-acetylated glucosamine). The two samples were recovered and analyzed by the two methods, and compared with the parent heparin. Calculations for the SAX-HPLC method required some adjustments to take into consideration the unknown peaks (at least 6) originating in these samples. A complete overview of results from these experiments can be found in Supplementary Table 9a. These results showed that the content of ΔIh from the SAX-HPLC method increases, as was expected (from 0.1 to 2.4 and 6.9%, respectively), but the contents of the disaccharide ΔIs (peak 14 in Table 2) and of almost all tetrasaccharides with glucosamines sulfated in position 3 are seriously affected by these treatments. Other disaccharides with N-sulfated glucosamines are affected too, even if to a lesser extent. Results from the HSQC method (Supplementary Table 10a), on the other hand, always show much higher contents of GlcNH_2_ compared to the SAX method. These data suggest that the attribute GlcNH_2_, from the disaccharide ΔIh, can be only partially quantified by the SAX-HPLC method because the enzymatic depolymerization originates many unidentified di- and tetra-saccharides that spread on the chromatogram. Therefore, accurate information on the content of N-desulfated glucosamine can be obtained from the HSQC method only.

The content of galacturonic acid (peaks 4 and 9 in Table 2, and GalA in Table 4), as a marker of alkaline treatments during the heparin extraction/purification steps (6, 32), is an important “process signature” able to identify batches produced by treatments that are too strong. The SAX-HPLC method is very sensitive to this feature: two disaccharides with a galacturonic structure were identified in almost all the considered heparin batches, with amounts of GalA ranging between the limit of detection (0.1%) and 6.2% (Supplementary Table 7). However, as discussed above, the HSQC method cannot quantify the content of galacturonic acid from the anomeric signals, which are embedded in the signal of 2-sulfate iduronic acids: the content of GalA is obtained by integration of the corresponding proton 5. This proton generates a broad and complex signal due to sequence effects, making its integration difficult, with a poor LOD/LOQ. The result of these considerations is that the HSQC method identifies GalA only in batches containing a high percentage of this residue and cannot provide accurate data (Figure 3C and Supplementary Table 7).

The epoxide residue has not been detected in any of the 33 heparin batches of our survey, either by SAX or HSQC, making the comparison of the two methods impossible. This residue is another marker of alkaline treatments, as it is considered the intermediate step of the process ending in galacturonic acid (6). To address this problem, a couple of specific experiments were designed: a heparin sample was deliberately over-stressed with a treatment with NaOH 0.4 M, for 1.0 and 2.5 h at 50°C. The two samples were recovered and analyzed by the two methods, in comparison with the parent heparin (subsection Uronic acid, 2,3-epoxide). The chromatograms of the SAX-HPLC method showed the appearance of two major and, at least, other 5 minor unknown, broad peaks, with λMax at 245 nm, when the typical λMax of all di- and oligosaccharides from the enzymatic cleavage is about 234 nm. On the contrary, the HSQC method could provide reliable data, through the identification and quantification of the typical epoxide residue signals (H2/C2 and H3/C3 at 3.71/54.5 and 3.78/53.5 ppm, respectively), undetectable in the parent heparin and present in amounts of 5.8 and 15.7% in the two samples, respectively. A complete overview of results from these experiments can be found in Supplementary Tables 9b, 10b. These results suggest that the epoxide, as a minor marker of 2-O-desulfation of heparin batches, can be quantified by the HSQC method only.

A further quality attribute of heparin, investigated in this study, is the “linkage region”, whose variants are useful tools for providing information about the methods used for heparin purification with regard to the oxidative stress. The presence of high amounts of the “native” linkage region (LR, the oligosaccharide -GlcA-Gal-Gal-Xyl-Serine) is a marker of a slight stress, while the presence of different oxidized variants (e.g., the oligosaccharide -GlcA-Gal-Gal-Xyl-CH_2_COOH) is a marker of strong treatments (7). Also in this case, the two methods do not provide the same kind of information: the SAX-HPLC method identifies only two major species (“native” LR and the oxidized species ΔUA-Gal-Gal-Xyl-CH_2_COOH), while the HSQC method detects all glucuronic acids linked to a galactose, including many other, more strongly oxidized, residues (-GlcA-Gal-remnant). The total amounts of linkage region detected by SAX-HPLC are much lower than those obtained by HSQC. This finding was expected, as the HSQC method detects a higher number of oxidized variants, with the only limit of the presence of glucuronic acid still linked to the first galactose. However, the very high content of LR found in many heparin batches (more than 5.0%, Supplementary Table 7), supports a possible over-estimation of the content of LR, native or oxidized, by the HSQC method. This over-estimation could be related to a higher “mobility” of the LR, which is not sulfated and located at the reducing end terminal of heparin chains. This “extra” mobility may induce a longer relaxation time compared to the other parts of the chain, corresponding to a minor loss of transverse relaxation time during the pulse sequence, with the consequent slight over-estimation of the volume of the H1/C1 GlcA-Gal signal. According to these findings, neither SAX-HPLC nor HSQC can provide accurate information on the LR content, but the comparison of HSQC data and the content of ΔUA-Gal-Gal-Xyl-Serine by SAX-HPLC can be still considered suitable for estimating the oxidative stress of heparin batches.

## Conclusions

This study, comparing the outcomes of SAX-HPLC and HSQC-NMR methods, proved to be very useful in investigating their accuracy, and their strong points or shortcomings. Though both SAX-HPLC and HSQC are unable to determine how the distinct clusters (e.g., clusters of N-acetylated or N-sulfated glucosamine) are distributed along the heparin chains, they provide a great amount of high-quality and complementary information on the building blocks composition of Heparin batches. Despite some minor differences, both methods provide a consistent comparison of heparin batches from the same or different manufacturers, suitable for keeping under control the quality of the drug and applicable to all the heparin types available on the market.

Some major structural differences can be observed using both techniques, i.e., sulfation level in position 2 of the uronic acid residues and in position 2 and 6 of glucosamine. On the other hand, the ratio between iduronic and glucuronic acid is obtained only by HSQC-NMR, whereas the ratio between the different antithrombin-binding pentasaccharide structures can be determined exclusively by the SAX-HPLC method, as tetrasaccharide sequences (15, 16). Many of the minor discrepancies between the two methods, as far as the content of some residues is concerned, can be related to the peculiarities of each method. For instance, the content of 6-O-sulfated glucosamine proved to be lower when calculated by HSQC due to the different relaxation properties of the sulfated and non-sulfated C6 group. On the other hand, sequences containing epoxide residues, induced by the alkaline treatment on the 2-sulfated-iduronic acid, cannot be cleaved by enzymes and the generated oligosaccharides are hardly detected by the SAX-HPLC method. Moreover, it was experimentally demonstrated that the molar absorption coefficients of the major Δ4-5 unsaturated heparin disaccharides are very similar and that the small differences observed for few disaccharides present in small amounts have no influence on the final results of the SAX method.

In conclusion, the results described in the present study demonstrated that both methods are sufficiently accurate to determine the fingerprint of heparin, making them suitable for monitoring the different steps of heparin manufacturing process as well as for ensuring the quality of the products on the market. Moreover, the combined use of these methods increases the number of detectable structural features useful also for differentiating between heparins of different animal and organ sources more effectively. Additionally, the application of chemometric analysis to the large set of data achievable from these methods is a promising tool for detecting structural anomalies and possible cross-species contamination.

## Author Contributions

LL, FS, MG, and AN contributed to planning and writing the paper. MM performed the NMR analyses. AP performed the SAX-HPLC analysis. AP and EU gave support for the SAX-HPLC data interpretation. EU performed the characterization of non-commercially available di- and oligosaccharides. All authors contributed to revision of the manuscript and read and approved the submitted version.

### Conflict of Interest Statement

FS, LL, and AP were employed by Opocrin S.p.A. The remaining authors declare that the research was conducted in the absence of any commercial or financial relationships that could be construed as a potential conflict of interest.
