# Supplementary material for: SAX-HPLC and HSQC NMR Spectroscopy: Orthogonal Methods for Characterizing Heparin Batches Composition
Source: Front Med (Lausanne). 2019 Apr 18;6:78. doi: 10.3389/fmed.2019.00078 (PMC6482219; doi:10.3389/fmed.2019.00078)
Supplement: Supplementary file 1 [file Data_Sheet_1.PDF]

Supplementary Material

SAX-HPLC and HSQC NMR spectroscopy: orthogonal methods for characterizing Heparin batches composition.

Franco Spelta*, Lino Liverani, Alessandra Peluso, Maria Marinozzi, Elena Urso, Marco Guerrini, Annamaria Naggi

*** Correspondence:** Dr. Franco Spelta
f.spelta@opocrin.it

1 Supplementary Data

S-Tx_1 Check of completeness of enzymatic digestion: check by Size Exclusion Chromatography

Fifteen samples from a random selection, which have been digested according to the standard procedure, were checked for absence of oligosaccharides longer than a tetrasaccharide, by UHP-SEC.

The digested samples (5 µL) were injected in a BEH 125 column (Waters), on a Acquity, H-Class UHPLC system (Waters). Mobile phase was ammonium acetate 0.1M, pH 6.0; flow rate 0.5 mL/min, column temperature 35 °C. Double detection by UV (234 nm) and Refractive Index, at 35 °C.

No peak, relevant to oligosaccharides longer than a tetrasaccharide, was detected by UV or RI, in any of the 15 samples; the estimated molar percentage of tetrasaccharides, by UV, was in the range between 3.0 and 5.7, in agreement with findings from the SAX-HPLC method, which showed a content of tetrasaccharides (linkage region and tetrasaccharides with a glucosamine 3-O-sulfated) in the range between 3.4 and 5.6 %; UHP-SEC data not shown.

2 Supplementary Figures and Tables

2.1 Supplementary Figures

S-Fig_1: Quantitative proton NMR spectrum of the ΔUA2S-GlcNS disaccharide. The H4 of the unsaturated residue was used for integration

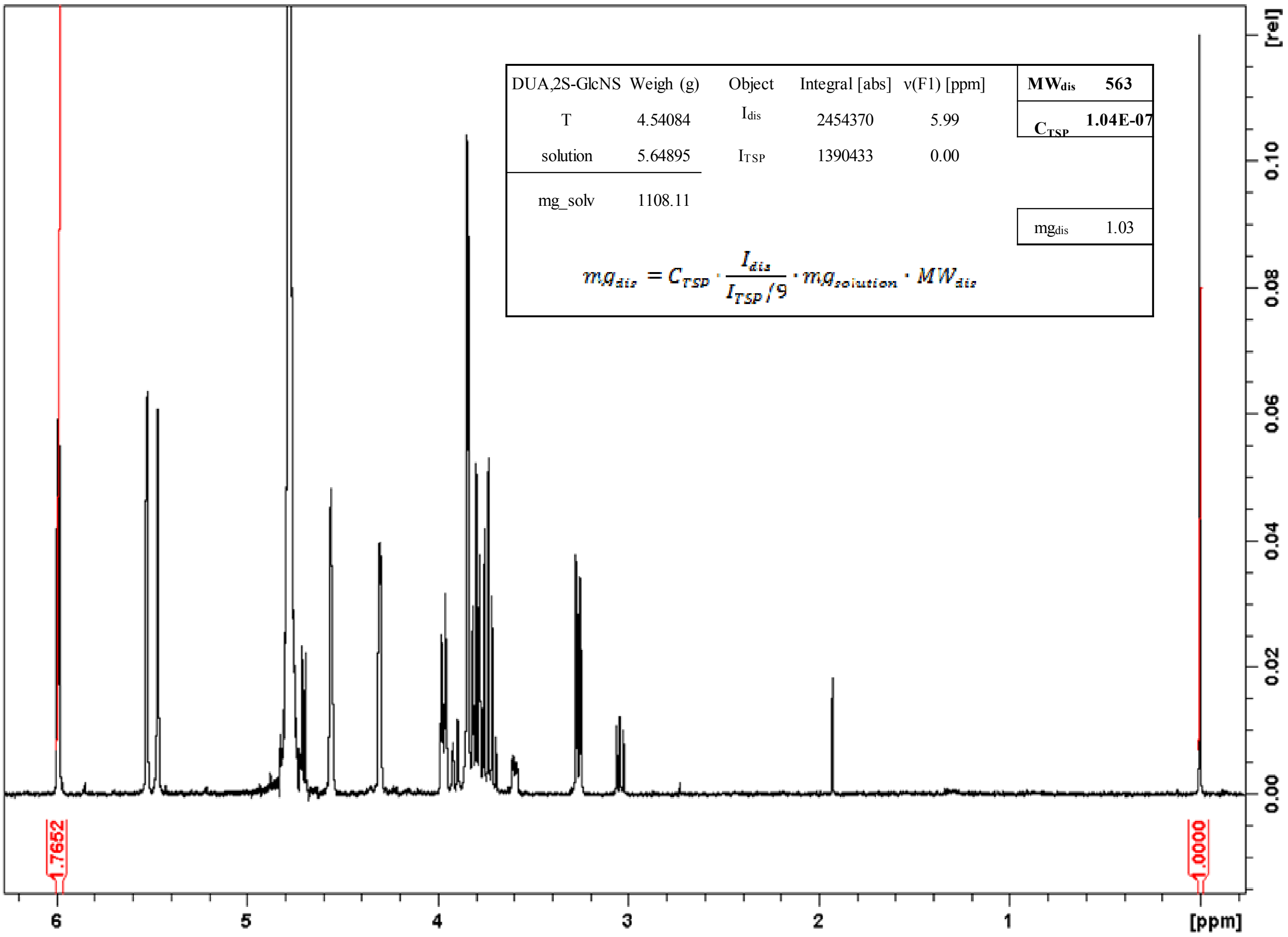

2.2 Supplementary Tables

S-Tab_1 Summary of the validation of the SAX-HPLC method

**Characteristics**	**Results**
Repeatability	RSD% for injection, in a range between 0.0 and 1.6
	RSD% for sample preparation, in a range between 0.3 and 2.0
	Test NOT passed by disaccharide ΔIh, peak **8** (RSD% 11.5)
Intermediate precision	RSD% in a range between 0.2 and 2.3
	Test NOT passed by disaccharide ΔIh, (RSD% 25.7)
LOD	0.1 %
LOQ	0.3 %

S-Tab_2 List of heparin batches compared in the study, with manufacturer identification and date of receipt of the sample

manufacturer - sample	***received in***	***manufacturer - sample***	***received in***
Man_A-1	04/2011	Man_E-1	02/2015
Man_A-2	04/2011	Man_E-2	06/2015
Man_A-3	04/2011	Man_E-3	09/2015
Man_A-4	04/2011	Man_F-1	02/2015
Man_A-5	04/2011	Man_F-2	06/2015
Man_B-1	12/2011	Man_G-1	07/2015
Man_B-2	03/2012	Man_G-2	07/2015
Man_B-3	05/2012	Man_H-1	02/2016
Man_B-4	11/2014	Man_H-2	02/2016
Man_B-5	06/2015	Man_H-3	02/2016
Man_C-1	06/2012	Man_H-4	02/2016
Man_D-1	01/2015	Man_H-5	02/2016
Man_D-2	06/2015	Man_H-6	02/2016
Man_D-3	08/2015	Man_H-7	02/2016
Man_D-4	08/2015	Man_H-8	02/2016
Man_D-5	08/2015		
Man_D-6	08/2015		
Man_D-7	08/2015		

S-Tab_3 Response factor for the typical disaccharides of heparin: concentration by proton NMR and response factor in SAX-HPLC

			Sample concentration by proton NMR		Response Factor in SAX-HPLC	
peak ID	disaccharides		mg/mL	µmol/mL	areaU / nmol inj.	ratio with respect to disacch. ΔIs
2	ΔUA-GlcNAc	ΔIVa	0.635	1.584	373331	0.94
5	ΔUA-GlcNS	ΔIVs	0.625	1.356	380489	0.96
6	ΔUA-GlcNAc,6S	ΔIIa	1.200	2.386	376153	0.95
7	ΔUA,2S-GlcNAc	ΔIIIa	0.760	1.511	389968	0.98
8	ΔUA,2S-GlcN,6S	ΔIh	0.665	1.181	374945	0.94
10	ΔUA-GlcNS,6S	ΔIIs	0.865	1.536	402969	1.01
11	ΔUA,2S-GlcNS	ΔIIIs	1.040	1.847	400054	1.01
12	ΔUA,2S-GlcNAc,6S	ΔIa	1.075	1.777	396696	1.00
14	ΔUA,2S-GlcNS,6S	ΔIs	0.955	1.436	397447	1.00

S-Tab_4 Raw data from the SAX-HPLC method: molar percentages of all identified residues

	Peak ID																		
manufacturer - sample	**p_01**	**p_02**	**p_03**	**p_04**	**p_05**	**p_06**	**p_07**	**p_08**	**p_09**	**p_10**	**p_11**	**p_12**	**p_13**	**p_14**	**p_15**	**p_16**	**p_17**	**p_18**	**p_19**
Man_A-1	0.7	3.2	0.3	0.3	2.9	2.8	1.6	0.1	1.2	10.3	7.9	1.4	0.5	64.3	2.1	0.2	0.2	0.1	0.2
Man_A-2	0.4	3.1	0.5	0.5	2.9	2.3	1.6	0.1	2.1	10.5	8.6	1.2	0.5	63.6	1.8	0.2	0.2	0.1	0.2
Man_A-3	0.3	2.9	0.4	0.7	3.0	2.6	1.6	0.1	2.2	11.1	8.0	1.2	0.4	62.8	1.9	0.2	0.2	0.1	0.2
Man_A-4	0.2	3.2	0.4	0.4	2.9	2.5	1.6	0.1	1.4	10.8	8.0	1.3	0.5	64.3	1.9	0.2	0.1	0.1	0.2
Man_A-5	0.4	3.0	0.4	0.5	2.9	2.4	1.6	0.1	1.9	10.8	8.3	1.2	0.5	63.6	1.8	0.2	0.2	0.2	0.2
Man_B-1	0.4	3.2	0.7	0.8	2.9	3.1	1.2	0.1	5.7	10.6	5.6	1.4	0.5	60.3	2.9	0.4	0.2	0.1	0.3
Man_B-2	0.4	3.6	0.6	0.2	2.9	3.1	1.4	0.0	1.2	11.3	7.0	1.4	0.5	62.8	2.5	0.4	0.2	0.2	0.3
Man_B-3	0.6	3.4	0.6	0.1	2.7	2.9	1.6	0.0	0.9	10.7	6.5	1.5	0.6	64.6	2.5	0.3	0.2	0.2	0.2
Man_B-4	0.8	3.2	0.6	0.1	2.9	2.6	1.7	0.1	0.5	10.6	7.4	1.3	0.6	64.7	2.2	0.3	0.2	0.1	0.2
Man_B-5	1.7	4.7	0.3	0.1	3.6	3.1	1.7	0.1	0.7	10.7	7.5	1.4	0.5	61.1	2.2	0.3	0.2	0.2	0.2
Man_C-1	0.3	3.1	0.6	0.3	2.9	2.9	1.5	0.0	1.5	11.1	6.7	1.5	0.5	63.8	2.4	0.3	0.2	0.2	0.2
Man_D-1	0.5	3.9	1.1	0.2	2.9	3.2	1.3	0.1	1.3	10.8	6.9	1.5	0.5	62.5	2.7	0.3	0.2	0.1	0.2
Man_D-2	0.3	4.8	1.2	0.2	3.6	3.1	1.4	0.0	1.4	10.8	7.3	1.3	0.5	61.1	2.3	0.3	0.1	0.2	0.2
Man_D-3	0.6	4.6	1.4	0.1	3.5	3.3	1.6	0.1	0.8	11.1	7.6	1.5	0.5	60.3	2.3	0.2	0.1	0.2	0.2
Man_D-4	0.8	5.7	0.2	0.2	3.7	3.2	1.4	0.0	1.3	9.7	7.6	1.2	0.4	61.5	2.2	0.4	0.3	0.1	0.3
Man_D-5	1.3	5.8	0.2	0.1	4.4	3.5	1.8	0.0	1.0	10.5	7.8	1.3	0.5	59.1	2.1	0.3	0.2	0.1	0.2
Man_D-6	0.8	4.3	0.1	0.2	3.5	3.2	1.4	0.0	1.5	10.7	7.4	1.3	0.5	62.0	2.2	0.3	0.2	0.2	0.3
Man_D-7	1.0	5.6	0.2	0.3	4.4	3.5	1.7	0.0	1.9	10.3	7.6	1.3	0.5	58.8	2.1	0.3	0.2	0.1	0.2

S-Tab_4 Raw data from the SAX-HPLC method: molar percentages of all identified residues - Continued

	Peak ID																		
manufacturer - sample	**p_01**	**p_02**	**p_03**	**p_04**	**p_05**	**p_06**	**p_07**	**p_08**	**p_09**	**p_10**	**p_11**	**p_12**	**p_13**	**p_14**	**p_15**	**p_16**	**p_17**	**p_18**	**p_19**
Man_E-1	1.4	3.8	1.0	0.0	2.7	3.0	1.7	0.1	0.2	10.5	7.3	1.4	0.5	63.5	2.3	0.2	0.2	0.2	0.2
Man_E-2	1.5	4.2	0.9	0.0	3.0	3.3	1.8	0.0	0.1	10.8	8.2	1.5	0.5	61.7	2.1	0.2	0.1	0.1	0.1
Man_E-3	1.1	4.2	0.9	0.0	3.1	3.4	1.9	0.0	0.1	10.3	8.2	1.5	0.5	62.2	2.1	0.2	0.1	0.1	0.2
Man_F-1	0.7	5.6	0.1	0.2	3.7	3.1	1.3	0.0	1.0	10.3	7.6	1.1	0.4	61.6	2.3	0.4	0.2	0.1	0.3
Man_F-2	0.7	5.4	0.1	0.3	4.3	3.5	1.6	0.0	2.2	10.9	7.6	1.3	0.5	58.7	2.1	0.3	0.1	0.1	0.2
Man_G-1	0.2	5.1	1.1	0.1	3.6	3.3	1.6	0.0	0.8	10.2	7.4	1.5	0.5	61.3	2.6	0.3	0.2	0.1	0.2
Man_G-2	1.2	5.6	1.1	0.1	4.0	3.2	1.6	0.0	0.5	10.8	7.5	1.4	0.5	59.6	2.3	0.3	0.2	0.1	0.2
Man_H-1	1.4	3.6	0.8	0.0	2.7	2.9	1.6	0.0	0.4	10.7	7.3	1.4	0.5	63.6	2.3	0.3	0.2	0.2	0.2
Man_H-2	1.5	4.2	0.9	0.0	3.0	3.4	1.9	0.0	0.1	10.3	8.2	1.5	0.5	61.9	2.1	0.2	0.2	0.1	0.2
Man_H-3	1.4	4.0	0.9	0.0	3.0	3.2	1.8	0.0	0.2	10.7	8.0	1.4	0.5	62.2	2.1	0.2	0.1	0.1	0.2
Man_H-4	1.1	3.9	1.2	0.0	2.8	3.1	1.7	0.0	0.1	10.6	7.4	1.5	0.5	63.2	2.2	0.2	0.2	0.1	0.2
Man_H-5	1.2	3.8	1.1	0.0	2.7	3.0	1.7	0.0	0.1	10.6	7.3	1.4	0.5	63.5	2.3	0.2	0.2	0.1	0.2
Man_H-6	1.5	4.2	0.9	0.0	3.0	3.3	1.8	0.0	0.1	10.9	8.1	1.4	0.5	61.6	2.1	0.2	0.2	0.1	0.2
Man_H-7	1.4	3.8	1.0	0.0	2.7	2.9	1.7	0.0	0.2	10.5	7.3	1.4	0.5	63.5	2.3	0.2	0.2	0.2	0.2
Man_H-8	0.8	4.0	1.5	0.1	2.9	3.3	1.8	0.0	0.3	10.6	7.9	1.5	0.5	62.0	2.2	0.3	0.2	0.1	0.2

S-Tab_5 Raw data from the HSQC method: molar percentages of all identified residues

S-Tab_5a Percentages of Glucosamine residues

	$GlcNH_2$,6x	GlcNS,3S,6x	GlcNAc,6x-GlcA	GlcNAc,6x-IdoA	GlcNS,6x-GlcA	GlcNS,6x-IdoA	GlcNS,6x-IdoA,2S	GlcNS,6x-GalA	GlcNS,6x-Epox	GlcNAc,6x-αRed	GlcNS,6x-αRed	GlcNx,6S
Man_A-1	1.0	4.4	9.3	0.7	9.8	7.2	64.5	1.5	< LOD	0.7	1.0	77.1
Man_A-2	1.3	4.1	11.8	< LOD	9.9	7.8	61.0	3.4	< LOD	< LOD	0.7	76.8
Man_A-3	1.3	4.1	10.0	0.6	9.8	9.2	60.3	3.6	< LOD	0.6	< LOD	77.0
Man_A-4	1.1	3.8	11.1	< LOD	11.0	7.0	62.3	3.2	< LOD	< LOD	< LOD	76.7
Man_A-5	1.1	4.1	9.8	< LOD	9.3	7.8	63.5	3.2	< LOD	0.8	< LOD	78.0
Man_B-1	< LOD	5.5	11.5	< LOD	9.0	9.9	59.1	4.2	< LOD	< LOD	0.8	81.1
Man_B-2	< LOD	5.4	13.2	< LOD	10.0	8.9	62.4	< LOD	< LOD	< LOD	< LOD	79.1
Man_B-3	0.7	5.6	13.7	< LOD	9.9	7.7	60.5	1.3	< LOD	< LOD	< LOD	79.8
Man_B-4	1.6	4.7	11.0	0.7	10.7	7.9	62.8	0.7	< LOD	< LOD	< LOD	78.5
Man_B-5	1.4	4.6	13.9	< LOD	9.2	8.5	61.0	1.2	< LOD	< LOD	< LOD	76.1
Man_C-1	< LOD	5.1	13.2	< LOD	10.1	8.7	59.6	3.2	< LOD	< LOD	< LOD	78.9
Man_D-1	1.2	4.7	12.8	< LOD	9.8	9.3	60.4	1.8	< LOD	< LOD	< LOD	79.2
Man_D-2	1.8	4.8	13.1	0.8	10.1	9.3	58.8	2.0	< LOD	< LOD	< LOD	76.6
Man_D-3	1.6	4.8	13.0	0.9	9.4	9.0	60.5	< LOD	< LOD	< LOD	0.8	74.7
Man_D-4	1.4	4.8	11.6	< LOD	10.3	8.7	59.9	1.0	< LOD	1.2	1.0	77.3
Man_D-5	1.6	4.4	12.6	0.8	10.8	7.8	59.1	1.5	< LOD	1.0	< LOD	73.8
Man_D-6	1.0	5.0	9.3	0.7	10.8	7.8	60.7	2.1	< LOD	1.0	1.7	78.0
Man_D-7	1.2	4.1	12.1	< LOD	10.3	8.8	58.8	2.0	< LOD	1.6	1.2	73.6
Man_E-1	< LOD	4.9	11.9	< LOD	9.5	8.5	64.4	< LOD	< LOD	0.8	< LOD	78.2
Man_E-2	0.7	4.4	12.1	1.0	9.7	8.1	62.8	< LOD	< LOD	0.9	< LOD	76.3
Man_E-3	< LOD	3.9	11.2	0.8	7.6	8.8	66.0	< LOD	< LOD	1.0	0.7	76.8

S-Tab_5a Percentages of Glucosamine residues - Continued

	GlcNH$_2$,6x	GlcNS,3S,6x	GlcNAc,6x-GlcA	GlcNAc,6x-IdoA	GlcNS,6x-GlcA	GlcNS,6x-IdoA	GlcNS,6x-IdoA,2S	GlcNS,6x-GalA	GlcNS,6x-Epox	GlcNAc,6x-αRed	GlcNS,6x-αRed	GlcNx,6S
Man_F-1	1.1	4.8	10.4	< LOD	10.5	7.6	59.2	3.3	< LOD	1.5	1.6	77.1
Man_F-2	1.5	4.3	12.7	< LOD	10.5	9.0	57.2	2.7	< LOD	1.3	0.9	74.7
Man_G-1	2.1	5.1	12.8	< LOD	8.0	7.6	63.9	1.3	< LOD	< LOD	< LOD	76.8
Man_G-2	1.8	4.9	14.3	0.8	8.3	8.8	61.1	< LOD	< LOD	< LOD	< LOD	75.0
Man_H-1	0.7	4.8	11.9	0.7	9.9	8.2	63.0	0.7	< LOD	< LOD	< LOD	78.4
Man_H-2	0.8	4.6	12.0	1.0	9.1	7.8	62.9	0.6	< LOD	1.1	< LOD	75.1
Man_H-3	< LOD	4.3	12.4	0.8	9.0	8.2	62.7	1.1	< LOD	0.9	0.7	76.1
Man_H-4	< LOD	4.3	12.6	0.7	8.9	7.6	64.3	1.0	< LOD	< LOD	< LOD	78.7
Man_H-5	< LOD	4.6	13.4	< LOD	9.5	8.6	63.9	< LOD	< LOD	< LOD	< LOD	77.4
Man_H-6	1.0	4.8	11.8	0.8	9.9	8.5	63.1	< LOD	< LOD	1.0	< LOD	76.1
Man_H-7	< LOD	4.6	12.4	0.8	9.6	8.0	63.9	< LOD	< LOD	< LOD	0.7	77.9
Man_H-8	< LOD	4.9	10.5	0.9	10.0	8.1	64.4	< LOD	< LOD	1.3	< LOD	76.8

xx.x: underscored figures are lower than LOQ

S-Tab_5b Percentages of uronic acid residues and sulfation degree

	GlcA-GlcNAc,6x	GlcA-GlcNS,6x	GlcA-GlcNS,3S,6x	GlcA,2S	IdoA-GlcNy	IdoA-GlcNy,6S	IdoA,2S-GlcNH$_2$,6x	IdoA,2S-GlcNy,3x,6x	GalA	Epox	LR	SO_3^-/COO^-
Man_A-1	5.2	5.8	2.2	< LOD	2.1	5.3	1.6	74.2	< LOD	< LOD	3.6	2.46
Man_A-2	5.9	6.0	2.2	< LOD	2.4	4.5	1.4	73.7	0.7	< LOD	3.4	2.43
Man_A-3	5.6	6.8	2.1	< LOD	2.3	4.9	1.4	73.2	1.3	< LOD	2.5	2.43
Man_A-4	6.4	6.4	1.9	< LOD	2.4	4.7	1.0	73.7	< LOD	< LOD	3.5	2.43
Man_A-5	4.6	6.3	2.3	< LOD	1.8	4.7	1.4	75.5	0.5	< LOD	2.8	2.47
Man_B-1	5.2	6.2	3.3	< LOD	2.1	6.4	< LOD	70.6	3.2	< LOD	2.9	2.46
Man_B-2	6.2	6.5	2.4	< LOD	2.6	6.4	< LOD	72.6	< LOD	< LOD	3.5	2.44
Man_B-3	5.2	5.8	3.1	< LOD	2.6	6.4	1.5	71.2	< LOD	< LOD	4.1	2.44
Man_B-4	5.2	6.4	2.4	< LOD	2.2	5.2	1.7	72.9	< LOD	< LOD	3.9	2.45
Man_B-5	6.5	6.0	2.6	< LOD	3.0	6.2	1.7	69.2	< LOD	< LOD	4.8	2.36
Man_C-1	6.8	6.1	2.5	< LOD	2.5	6.1	< LOD	72.3	< LOD	< LOD	3.6	2.43
Man_D-1	5.4	6.3	2.6	< LOD	2.3	6.7	1.7	70.6	< LOD	< LOD	4.3	2.42
Man_D-2	5.6	6.0	2.8	< LOD	2.7	6.1	1.7	71.0	< LOD	< LOD	4.2	2.38
Man_D-3	6.2	6.7	2.5	< LOD	2.3	6.0	2.2	69.3	< LOD	< LOD	4.9	2.35
Man_D-4	6.2	5.7	2.2	< LOD	2.7	5.8	1.5	73.8	< LOD	< LOD	2.1	2.43
Man_D-5	7.0	6.2	2.5	< LOD	3.0	5.9	1.3	70.2	< LOD	< LOD	3.9	2.34
Man_D-6	5.4	6.8	3.1	< LOD	2.4	6.1	1.3	72.3	< LOD	< LOD	2.6	2.45
Man_D-7	7.0	6.4	2.7	< LOD	3.1	6.3	1.5	69.9	< LOD	< LOD	3.1	2.34
Man_E-1	5.1	5.6	2.8	< LOD	2.5	5.8	0.8	71.9	< LOD	< LOD	5.5	2.43
Man_E-2	5.1	6.3	2.2	< LOD	2.8	6.3	1.0	71.1	< LOD	< LOD	5.2	2.38
Man_E-3	4.7	6.7	2.1	< LOD	2.6	6.4	0.6	71.5	< LOD	< LOD	5.4	2.40

S-Tab_5b Percentages of uronic acid residues and sulfation degree - Continued

	GlcA-GlcNAc,6x	GlcA-GlcNS,6x	GlcA-GlcNS,3S,6x	GlcA,2S	IdoA-GlcNy	IdoA-GlcNy,6S	IdoA,2S-GlcNH_2,6x	IdoA,2S-GlcNy,3x,6x	GalA	Epox	LR	SO_3^-/COO^-
Man_F-1	6.1	6.0	1.8	< LOD	2.7	6.1	1.4	73.7	< LOD	< LOD	2.3	2.44
Man_F-2	6.3	7.0	2.7	< LOD	3.0	6.7	1.6	69.1	0.7	< LOD	3.0	2.34
Man_G-1	5.3	6.2	2.8	< LOD	2.4	6.4	1.9	69.5	< LOD	< LOD	5.6	2.39
Man_G-2	5.8	6.5	3.2	< LOD	3.0	6.2	1.6	68.0	< LOD	< LOD	5.8	2.33
Man_H-1	4.6	6.2	2.4	< LOD	2.3	6.0	< LOD	73.7	< LOD	< LOD	4.9	2.44
Man_H-2	5.0	6.2	2.3	< LOD	2.9	6.1	< LOD	72.4	< LOD	< LOD	5.2	2.37
Man_H-3	5.7	5.8	2.3	< LOD	2.5	6.2	< LOD	71.8	< LOD	< LOD	5.7	2.38
Man_H-4	5.4	5.6	2.5	< LOD	2.4	5.7	< LOD	73.2	< LOD	< LOD	5.4	2.42
Man_H-5	6.6	6.6	2.2	< LOD	2.8	6.1	< LOD	70.4	< LOD	< LOD	5.4	2.39
Man_H-6	4.5	6.4	1.8	< LOD	2.4	6.3	1.0	72.3	< LOD	< LOD	5.3	2.39
Man_H-7	4.3	5.5	2.9	< LOD	2.7	5.5	< LOD	73.5	< LOD	< LOD	5.7	2.43
Man_H-8	5.8	6.7	2.3	< LOD	2.6	6.1	0.7	71.5	< LOD	< LOD	4.5	2.41

xx.x: underscored figures are lower than LOQ

S-Tab_6 **Formulas for adjusting and combining raw data from the SAX-HPLC and the HSQC-NMR methods into 11 heparin attributes. All attributes are reported as percentages, with the exception of the ratio sulfate to carboxylate ions (SO_3^- /COO^-).**

For this exercise, raw data (molar percentages) from the SAX method were rearranged according to formulas that summarize this process: first, all undigested tetrasaccharides are taken into consideration as a couple of disaccharides and the contents of the same disaccharide are summed up, so that the sum of all building blocks (as percentages) becomes higher than 100. All percentages are thereafter recalculated, taking into account the "additional" disaccharides obtained from this step. Figures from this new set of data are then combined according to the relevant formula to make up the results for the different attributes.
Raw data from the HSQC method were rearranged in a slightly different way: in this case, the integral values of the relevant signals were first normalized with reference to the sum of volumes of signals corresponding to the same monosaccharide type (glucosamines or uronic acids). Figures from these calculations were then combined according to the relevant formula to make up the results for the same 11 attributes.
Formulas used for these two processes are shown in the table.

Heparin attribute	Formula for combining the SAX data	Formula for combining the HSQC data
GlcNS	[p_04 + p_05 + p_09 + p_10 + p_11 + p_13 + p_14 + p_15 + p_16*2 + p_17 + p_18 + p_19*2] / [100 + sum_tetra]	GlcNS,3S,6x + (GlcNS,6x-GlcA) + (GlcNS,6x-IdoA) + (GlcNS,6x-IdoA,2S) + (GlcNS,6x-GalA) + (GlcNS,6x-Epox) + (GlcNS,6x-αred)
GlcNAc	[p_02 + p_06 + p_07 + p_12 + p_13 + p_15 + p_18] / [100 + sum_tetra]	(GlcNAc,6x-GlcA) + (GlcNAc,6x-IdoA) + (GlcNAc,6x-αred)
GlcNx,6S	[p_06 + p_08 + p_09 + p_10 + p_12 + p_13 + p_14 + p_15*2 + p_16*2 + p_17 + p_18*2 + p_19*2] / [100 + sum_tetra]	GlcNy,6S
GlcNS,3S,6x	cannot be determined; estimated as [p_13 + p_15 + p_16 + p_17 + p_18 + p_19] / [100 + sum_tetra]	GlcNS,3S,6x
GlcA-GlcNS,3S,6x	[p_13 + p_15 + p_16 + p_18 + p_19] / [100 + sum_tetra]	GlcA-GlcNS,3S,6x
$GlcNH_2$	[p_08] / [100 + sum_tetra]	$GlcNH_2$,6x
IdoA,2S	[p_07 + p_08 + p_11 + p_12 + p_14 + p_17 + p_18 + p_19] / [100 + sum_tetra]	(IdoA,2S-GlcNy,3x,6x) + (IdoA,2S-$GlcNH_2$,6x)
GalA	[p_04 + p_09] / [100 + sum_tetra]	GalA
Epox	cannot be determined	Epox
Linkage region	[p_01 + p_03] / [100 + sum_tetra]	LR
SO_3^-/COO^-	[GlcNS + GlcNx,6S + GlcA-GlcNS,3S,6x + IdoA,2S] / 100	[(GlcNS,6x-GlcA) + (GlcNS,6x-IdoA) +(GlcNS,6x-IdoA,2S) + (GlcNS,6x-GalA) + (GlcNS,6x-Epox) + (GlcNS,6x-αred) + 2x GlcNS,3S,6x + GlcNy,6S + (IdoA,2S- GlcNy,3x,6x) + (IdoA,2S-$GlcNH_2$,6x) + GlcA,2S] / 100

Sum_tetra = [p_13 + p_15 + p_16 + p_18 + p_19]

S-Tab_7 Comparison of results from the two methods for the 11 specific heparin attributes, after the required calculations.

Manufacturer - sample	GlcNS		IdoA,2S		GlcNx,6S		GlcNAc		GlcNS,3S,6x		GlcA-GlcNS,3S,6x		$GlcNH_2$		GalA		LR		SO_3^-/COO^-	
	SAX HPLC	HSQC	SAX HPLC	HSQC	SAX HPLC	HSQC	SAX HPLC	HSQC	SAX HPLC	HSQC	SAX HPLC	HSQC	SAX HPLC	HSQC	SAX HPLC	HSQC	SAX HPLC	HSQC	SAX HPLC	HSQC
Man_A-1	87.8	88.4	73.5	75.8	83.3	77.1	11.2	10.6	3.2	4.4	3.0	2.2	0.1	1.0	1.4	0.0	1.0	3.7	2.51	2.46
Man_A-2	89.0	86.9	73.5	75.0	82.5	76.8	10.1	11.8	2.8	4.1	2.6	2.2	0.0	1.3	2.5	0.7	0.9	3.5	2.51	2.43
Man_A-3	88.8	87.5	72.2	74.6	83.0	77.0	10.5	11.2	2.9	4.1	2.7	2.1	0.0	1.3	2.8	1.3	0.7	2.6	2.50	2.43
Man_A-4	88.6	87.8	73.6	74.7	83.3	76.7	10.8	11.1	2.9	3.8	2.8	1.9	0.1	1.1	1.7	0.0	0.6	3.7	2.51	2.43
Man_A-5	88.9	88.2	73.3	76.9	83.0	78.0	10.3	10.6	2.9	4.1	2.8	2.3	0.0	1.1	2.3	0.5	0.8	2.9	2.51	2.47
Man_B-1	87.1	88.5	66.4	70.6	85.4	81.1	11.8	11.5	4.1	5.5	3.9	3.3	0.0	0.0	6.2	3.2	1.0	3.1	2.47	2.46
Man_B-2	86.7	86.8	70.5	72.6	84.0	79.1	12.3	13.2	3.9	5.4	3.7	2.4	0.1	0.0	1.3	0.0	1.0	3.7	2.49	2.44
Man_B-3	86.7	85.5	72.0	72.7	84.6	79.8	12.2	13.7	3.8	5.6	3.7	3.1	0.0	0.7	1.0	0.0	1.1	4.2	2.51	2.44
Man_B-4	87.3	86.7	73.3	74.7	83.4	78.5	11.3	11.8	3.4	4.7	3.2	2.4	0.0	1.6	0.6	0.0	1.3	4.0	2.51	2.45
Man_B-5	84.7	84.7	69.9	70.9	80.5	76.1	13.2	13.9	3.3	4.6	3.2	2.6	0.1	1.4	0.8	0.0	2.0	5.1	2.42	2.36
Man_C-1	87.4	86.8	71.4	72.3	84.6	78.9	11.7	13.2	3.7	5.1	3.5	2.5	0.1	0.0	1.7	0.0	0.9	3.8	2.51	2.43
Man_D-1	85.8	86.0	70.2	72.3	83.4	79.2	12.6	12.8	3.8	4.7	3.6	2.6	0.1	1.2	1.4	0.0	1.5	4.5	2.47	2.42
Man_D-2	85.4	84.3	69.2	72.6	81.4	76.6	13.2	13.9	3.5	4.8	3.4	2.8	0.0	1.8	1.5	0.0	1.4	4.3	2.43	2.38
Man_D-3	84.6	84.5	69.2	71.5	80.8	74.7	13.5	13.9	3.5	4.8	3.3	2.5	0.1	1.6	0.9	0.0	1.9	5.0	2.41	2.35
Man_D-4	85.4	85.7	69.9	75.3	80.8	77.3	13.6	12.9	3.6	4.8	3.3	2.2	0.0	1.4	1.4	0.0	0.9	2.1	2.43	2.43
Man_D-5	84.0	84.0	68.3	71.5	78.8	73.8	14.6	14.4	3.2	4.4	3.0	2.5	0.0	1.6	1.1	0.0	1.4	4.0	2.37	2.34
Man_D-6	86.5	88.1	70.3	73.6	82.5	78.0	12.6	10.9	3.5	5.0	3.3	3.1	0.0	1.0	1.7	0.0	0.9	2.7	2.46	2.45
Man_D-7	84.4	85.1	67.8	71.4	79.3	73.6	14.4	13.7	3.2	4.1	3.1	2.7	0.0	1.2	2.2	0.0	1.2	3.1	2.38	2.34
Man_E-1	85.2	87.4	72.1	72.7	82.2	78.2	12.5	12.6	3.4	4.9	3.3	2.8	0.0	0.0	0.1	0.0	2.3	5.8	2.46	2.43
Man_E-2	84.7	85.2	71.3	72.1	80.6	76.3	13.0	14.1	3.1	4.4	3.0	2.2	0.1	0.7	0.1	0.0	2.3	5.4	2.43	2.38
Man_E-3	84.8	87.0	71.9	72.1	80.7	76.8	13.2	13.0	3.1	3.9	3.0	2.1	0.0	0.0	0.1	0.0	2.0	5.7	2.44	2.40

S-Tab_7 - Comparison of results from the two methods for the 11 specific heparin attributes, after the required calculations - Continued

Manufacturer - sample	GlcNS		IdoA,2S		GlcNx,6S		GlcNAc		GlcNS,3S,6x		GlcA-GlcNS,3S,6x		$GlcNH_2$		GalA		LR		SO_3^-/COO^-	
	SAX HPLC	HSQC	SAX HPLC	HSQC	SAX HPLC	HSQC	SAX HPLC	HSQC	SAX HPLC	HSQC	SAX HPLC	HSQC	SAX HPLC	HSQC	SAX HPLC	HSQC	SAX HPLC	HSQC	SAX HPLC	HSQC
Man_F-1	85.8	87.0	69.8	75.0	81.1	77.1	13.5	11.9	3.6	4.8	3.4	1.8	0.0	1.1	1.2	0.0	0.7	2.3	2.44	2.44
Man_F-2	85.1	84.6	67.5	70.7	80.0	74.7	14.1	14.0	3.2	4.3	3.0	2.7	0.0	1.5	2.5	0.7	0.8	3.1	2.39	2.34
Man_G-1	84.6	85.1	69.7	71.5	81.2	76.8	14.1	12.8	3.8	5.1	3.6	2.7	0.0	2.1	0.8	0.0	1.3	5.8	2.43	2.39
Man_G-2	83.6	83.1	68.2	69.6	79.2	75.0	14.3	15.1	3.4	4.9	3.3	3.2	0.0	1.8	0.6	0.0	2.2	6.0	2.38	2.33
Man_H-1	85.8	86.7	71.9	73.7	82.6	78.4	12.1	12.6	3.5	4.8	3.3	2.4	0.0	0.7	0.4	0.0	2.1	5.1	2.47	2.44
Man_H-2	84.5	85.1	71.7	72.4	80.4	75.1	13.2	14.1	3.1	4.6	3.0	2.3	0.0	0.8	0.1	0.0	2.3	5.3	2.43	2.37
Man_H-3	85.0	85.9	71.6	71.8	80.9	76.1	12.7	14.1	3.1	4.3	3.0	2.3	0.0	0.0	0.2	0.0	2.3	5.8	2.44	2.38
Man_H-4	85.2	86.1	71.9	73.2	82.1	78.7	12.6	13.3	3.4	4.3	3.2	2.5	0.0	0.0	0.1	0.0	2.2	5.6	2.46	2.42
Man_H-5	85.3	86.6	72.0	70.4	82.1	77.4	12.5	13.4	3.5	4.6	3.3	2.2	0.0	0.0	0.1	0.0	2.3	5.7	2.46	2.39
Man_H-6	84.7	85.3	71.2	73.3	80.6	76.1	13.0	13.7	3.2	4.8	3.0	1.8	0.0	1.0	0.1	0.0	2.3	5.5	2.43	2.39
Man_H-7	85.4	86.8	72.1	73.5	82.2	77.9	12.4	13.2	3.4	4.6	3.2	2.9	0.0	0.0	0.2	0.0	2.3	5.9	2.47	2.43
Man_H-8	84.9	87.3	71.3	72.2	81.1	76.8	12.9	12.7	3.2	4.9	3.1	2.3	0.0	0.0	0.4	0.0	2.2	4.7	2.44	2.41
Min	83.6	83.1	66.4	69.6	78.8	73.6	10.1	10.6	2.8	3.8	2.6	1.8	0.0	0.0	0.1	0.0	0.6	2.1	2.4	2.3
Max	89.0	88.5	73.6	76.9	85.4	81.1	14.6	15.1	4.1	5.6	3.9	3.3	0.1	2.1	6.2	3.2	2.3	6.0	2.5	2.5
Mean	85.9	86.2	70.9	72.8	81.9	77.1	12.6	12.9	3.4	4.6	3.2	2.5	0.0	0.9	1.2	0.2	1.5	4.4	2.5	2.4
St. dev.	1.49	1.36	1.85	1.72	1.64	1.70	1.17	1.17	0.31	0.44	0.30	0.38	0.03	0.66	1.21	0.62	0.62	1.20	0.04	0.04

S-Tab_8 Comparison of GlcNx,6S data from the SAX, the HSQC and the carbon NMR methods.

sample	SAX	HSQC	^{13}C
Man_A-3	83.0	77.0	82.8
Man_A-4	83.3	76.7	83.9
Man_B-1	85.4	81.1	86.2
Man_B-3	84.6	79.8	85.3
Man_B-4	83.4	78.5	84.7
Man_G-2	79.2	75.0	81.1

S-Tab_9 Complete overview of SAX results from samples with increased content of N-desulfated glucosamine ($GlcANH_2$) and epoxide (Epox).

All raw data from these samples have been recalculated to obtain more simple tables:

i) all tetrasaccharides are taken into consideration as a couple of disaccharides and the contents of the same disaccharides are summed up

ii) the values of all residues, both summed up and not modified, are recalculated by dividing each one by the same factor used in S-Tab_6 ([100 + sum_tetra])

S-Tab_9a: Samples with increased content of N-desulfated glucosamine: molar percentages

	Sample	ΔGlu-Gal-Gal-Xyl-Ser	ΔIVa	**Unk_1N**	**Unk_2N**	**Unk_3N**	ΔGlu-Gal-Gal-Xyl-CH_2COOH	ΔIVs gal	ΔIVs	ΔIIa	ΔIIIa	**Unk_4N**	ΔIh	ΔIIs gal	ΔIIs	ΔIIIs	**Unk_5N**	ΔIa	$IVs_{glu(3S)}$	ΔIs	**Unk_6N**	$IIs_{glu(3S)}$	ΔIs(3S)		***sumUnk_xN***
	peak ID	1	2				3	4	5	6	7		8	9	10	11		12		14			17		
parent Hep.	from Man_H	0.4	3.9	0.0	0.0	0.0	0.5	0.1	3.0	6.0	1.5	0.0	0.1	0.9	11.3	7.2	0.0	1.6	0.4	59.9	0.0	3.1	0.1		
N-desulf.	N-deS_1	0.5	4.0	**0.7**	**1.0**	**0.1**	0.5	0.1	3.1	5.6	1.6	**0.4**	2.4	0.9	11.1	6.9	**0.0**	1.7	0.3	56.9	**0.1**	2.1	0.1		**2.4**
	N-deS_2	0.5	4.3	**2.0**	**1.8**	**0.4**	0.6	0.1	3.1	5.2	1.7	**0.8**	6.9	0.8	10.6	6.2	**0.2**	1.8	0.1	51.6	**0.3**	1.0	0.0		**5.5**

Unk_xN: unknown peak, typical of samples with increased content of glucosamine non N-sulfated, non N-acetylated. The results of this table include the unknown peaks, and the sum of all unknown residues is calculated as “sum Unk-xN”

S-Tab_9b: Samples with increased content of epoxide on the iduronic acid: molar percentages

	Sample	Glu-Gal-Gal-Xyl-Ser	ΔIVa	**Unk_1E**	**Unk_2E**	Glu-Gal-Gal-Xyl-CH2COOH	Unk_3E	Unk_4E	ΔIVsgal	ΔIVs	ΔIIa	Unk_5E	ΔIIIa	ΔIh	ΔIIsgal	ΔIIs	ΔIIIs	ΔIa	Unk_6E	**Unk_7E**	$IVs_{glu(3S)}$	ΔIs	$IIs_{glu(3S)}$	$\Delta Is_{(3S)}$	***sumUnk_xE***
	peak ID	1	2			3			4	5	6		7	8	9	10	11	12				14		17	
parent Hep.	from Man_	0.4	3.9	0.0	0.0	0.5	0.0	0.0	0.1	3.0	6.0	0.0	1.5	0.1	0.9	11.3	7.2	1.6	0.0	0.0	0.4	59.9	3.1	0.1	
epox.	epox_1	0.4	3.9	0.1	0.2	0.8	0.6	0.7	0.1	3.2	6.5	0.2	1.3	0.1	1.2	11.9	5.6	1.5	0.2	0.2	0.4	57.6	3.4	0.1	**2.1**
	epox_2	0.5	4.2	0.2	0.6	0.9	2.1	2.1	0.2	3.5	7.0	0.4	0.8	0.1	2.0	13.0	3.6	1.3	0.3	0.5	0.5	52.7	3.5	0.1	**6.2**
							Fused peaks: Unk_3E very broad. Special calculations applied																		

Unk_xE: unknown peak, typical of samples with increased content of uronic acid, 2-3 epoxide. The results of this table include the unknown peaks, and the sum of all unknown residues is calculated as "sum Unk-xE"

S-Tab_10 Complete overview of HSQC results from samples with increased content of N-desulfated glucosamine ($GlcANH_2$) and epoxide (Epox). No adjustment to the calculation method is required.

S-Tab_10a: Samples with increased content of N-desulfated glucosamine: molar percentages

Glucosamines	Sample	$GlcNH_2$,6x	GlcNS,3S,6x	GlcNAc,6x-GlcA	GlcNAc,6x-IdoA	GlcNS,6x-GlcA	GlcNS,6x-IdoA	GlcNS,6x-IdoA2S	GlcNS,6x-GalA	GlcNS,6x-Epox	GlcNAc,6x αRed	GlcNS,6x αRed	GlcNy,6S
parent Hep.		1.5	5.4	13.2	< LOD	9.9	9.4	58.3	1.3	< LOD	1.0	1.0	83.3
N-desulf.	N-deS_1	6.1	3.7	12.1	< LOD	10.8	9.8	58.2	< LOD	< LOD	1.0	< LOD	83.6
	N-deS_2	15.0	1.0	13.4	0.8	9.3	10.3	51.8	< LOD	< LOD	< LOD	< LOD	83.1

Uronic acids	Sample	GlcA-GlcNAc,6x	GlcA-GlcNS,6x	GlcA-GlcNS,3S,6x	GlcA,2S	IdoA-GlcNy	IdoA-GlcNy,6S	IdoA,2S-$GlcNH_2$,6x	IdoA,2S-GlcNy,3x,6x	GalA	Epox	LR
parent Hep.		6.2	8.1	3.4	< LOD	3.8	6.7	1.9	66.4	< LOD	< LOD	3.5
N-desulf.	N-deS_1	6.7	7.7	2.6	0.7	3.0	7.9	6.4	61.4	< LOD	< LOD	3.6
	N-deS_2	6.1	8.3	3.4	< LOD	3.0	7.4	13.4	55.1	< LOD	< LOD	3.2

XX.X: underscored figures are lower than LOQ

S-Tab_10b: Samples with increased content of epoxide on the iduronic acid: molar percentages

Glucosamines	Sample	GlcNH$_2$,6x	GlcNS,3S,6x	GlcNAc,6x-GlcA	GlcNAc,6x-IdoA	GlcNS,6x-GlcA	GlcNS,6x-IdoA	GlcNS,6x-IdoA2S	GlcNS,6x-GalA	GlcNS,6x-Epox	GlcNAc,6x αRed	GlcNS,6x αRed	GlcNy,6S
parent Hep.		1.5	5.4	13.2	< LOD	9.9	9.4	58.3	1.3	< LOD	1.0	1.0	83.3
epox.	epox_1	1.5	4.9	13.4	< LOD	10.5	9.8	52.8	1.2	6.1	< LOD	< LOD	83.1
	epox_2	1.2	4.9	13.4	< LOD	10.1	8.9	44.0	1.7	15.9	< LOD	< LOD	83.9

Uronic acids	Sample	GlcA-GlcNAc,6x	GlcA-GlcNS,6x	GlcA-GlcNS,3S,6x	GlcA,2S	IdoA-GlcNy	IdoA-GlcNy,6S	IdoA,2S-GlcNH$_2$,6x	IdoA,2S-GlcNy,3x,6x	GalA	Epox	LR
parent Hep.		6.2	8.1	3.4	< LOD	3.8	6.7	1.9	66.4	< LOD	< LOD	3.5
epox.	epox_1	6.6	6.8	3.0	< LOD	3.4	7.3	1.8	61.9	< LOD	5.8	3.3
	epox_2	6.4	8.1	2.9	0.8	3.2	7.3	1.1	50.3	1.1	15.7	3.2

XX.X: underscored figures are lower than LOQ
